# Linguistic and ontological challenges of multiple domains contributing to transformed health ecosystems

**DOI:** 10.3389/fmed.2023.1073313

**Published:** 2023-03-15

**Authors:** Markus Kreuzthaler, Mathias Brochhausen, Cilia Zayas, Bernd Blobel, Stefan Schulz

**Affiliations:** ^1^Institute for Medical Informatics, Statistics and Documentation, Medical University of Graz, Graz, Austria; ^2^Department of Biomedical Informatics, University of Arkansas for Medical Sciences, Little Rock, AR, United States; ^3^Medical Faculty, University of Regensburg, Regensburg, Germany; ^4^eHealth Competence Center Bavaria, Deggendorf Institute of Technology, Deggendorf, Germany; ^5^First Medical Faculty, Charles University Prague, Prague, Czechia; ^6^Averbis GmbH, Freiburg, Germany

**Keywords:** natural language processing, electronic health records, precision medicine, biomedical semantics, formal ontologies, terminologies

## Abstract

This paper provides an overview of current linguistic and ontological challenges which have to be met in order to provide full support to the transformation of health ecosystems in order to meet precision medicine (5 PM) standards. It highlights both standardization and interoperability aspects regarding formal, controlled representations of clinical and research data, requirements for smart support to produce and encode content in a way that humans and machines can understand and process it. Starting from the current text-centered communication practices in healthcare and biomedical research, it addresses the state of the art in information extraction using natural language processing (NLP). An important aspect of the language-centered perspective of managing health data is the integration of heterogeneous data sources, employing different natural languages and different terminologies. This is where biomedical ontologies, in the sense of formal, interchangeable representations of types of domain entities come into play. The paper discusses the state of the art of biomedical ontologies, addresses their importance for standardization and interoperability and sheds light to current misconceptions and shortcomings. Finally, the paper points out next steps and possible synergies of both the field of NLP and the area of Applied Ontology and Semantic Web to foster data interoperability for 5 PM.

## 1. Background

Managing healthcare transformation towards personalized, preventive, predictive, and participative precision medicine (5 PM) is the background of a series of contributions for a broad audience [see the introductory paper to this Special Issue (1)], among which this paper highlights the role of language, semantics and standards for 5 PM. It intends to support the understanding of crucial notions in a field known as Biomedical Semantics.

5 PM considers individual health conditions, genetic and genomic dispositions in personal, social, occupational, environmental and behavioral contexts. The goal is to transform health and social care by fully understanding disease mechanisms and by turning health and social care from reactive to proactive. The current healthcare system transformations aiming at 5 PM medicine are supported by a broad range of technologies, with data-centered approaches playing a crucial role. Other than with clinical trials, quality data are intended not only to be collected and analyzed for a specific purpose but during the whole care process within a health ecosystem, i.e., a network of all relevant interconnected entities ranging from patients and carers to diagnostic and care processes targeting clinical conditions, pathogens, devices and being reflected by an ever-increasing amount of data.

The implementation of 5 PM involves multiple domains and disciplines with their specific objectives and perspectives, using a broad range of methodologies, educational backgrounds, skills and experiences as well as a broad range of resources. The technologies to be deployed range from wearable and implantable micro- and nanotechnologies, biomolecular analytical techniques such as the family of OMICS technologies, up to super- and quantum-computing and big data analytics. Many of these technologies only unfold their potential if rooted in semantic resources like terminologies, ontologies and information models as core requirements for data standardization and interoperability.

The challenge is not only to understand the world of sciences and practices contributing to 5 PM, but also to formally and consistently represent it, i.e., of multidisciplinary and dynamic systems in variable context. Thus, mapping and harmonization data and processes among the different disciplines, methodologies, perspectives, intentions, languages, etc. must be supported. This is bound to the advancement of communication and cooperation between the business actors from data to concept and knowledge levels, in order to provide high-quality integration and interoperability between and within health ecosystems. Consequently, knowledge representation (KR) and knowledge management (KM) are crucial for the transformation of health and social care.

KR and KM happen at three levels: (a) the epistemological level of domain-specific modeling; (b) the notation level of formalization and domain representation; (c) the processing level of implementations. The different levels are represented by languages of different abstraction and processability.

Like specialized dictionaries provide words that constitute textual expressions in a certain field of interest, domain ontologies provide the building blocks for the construction of knowledge in that domain, in order to support representation and communication. For enabling interoperability between different stakeholder perspectives as well as logical deduction and machine processing, we have to advance ontologies to become a repository of representational units for precise descriptions of classes of domain entities in logic-based languages (2). In order to bridge to the way humans communicate, in a variety of natural languages and domain-specific sublanguages, the entities of meaning, as collected and defined within ontologies, must be linked to collections of natural language terms, i.e., domain vocabularies. More information on the health and social care transformation and the challenges to properly model the systems can be found in (1, 3, 4).

**Figure 1:**
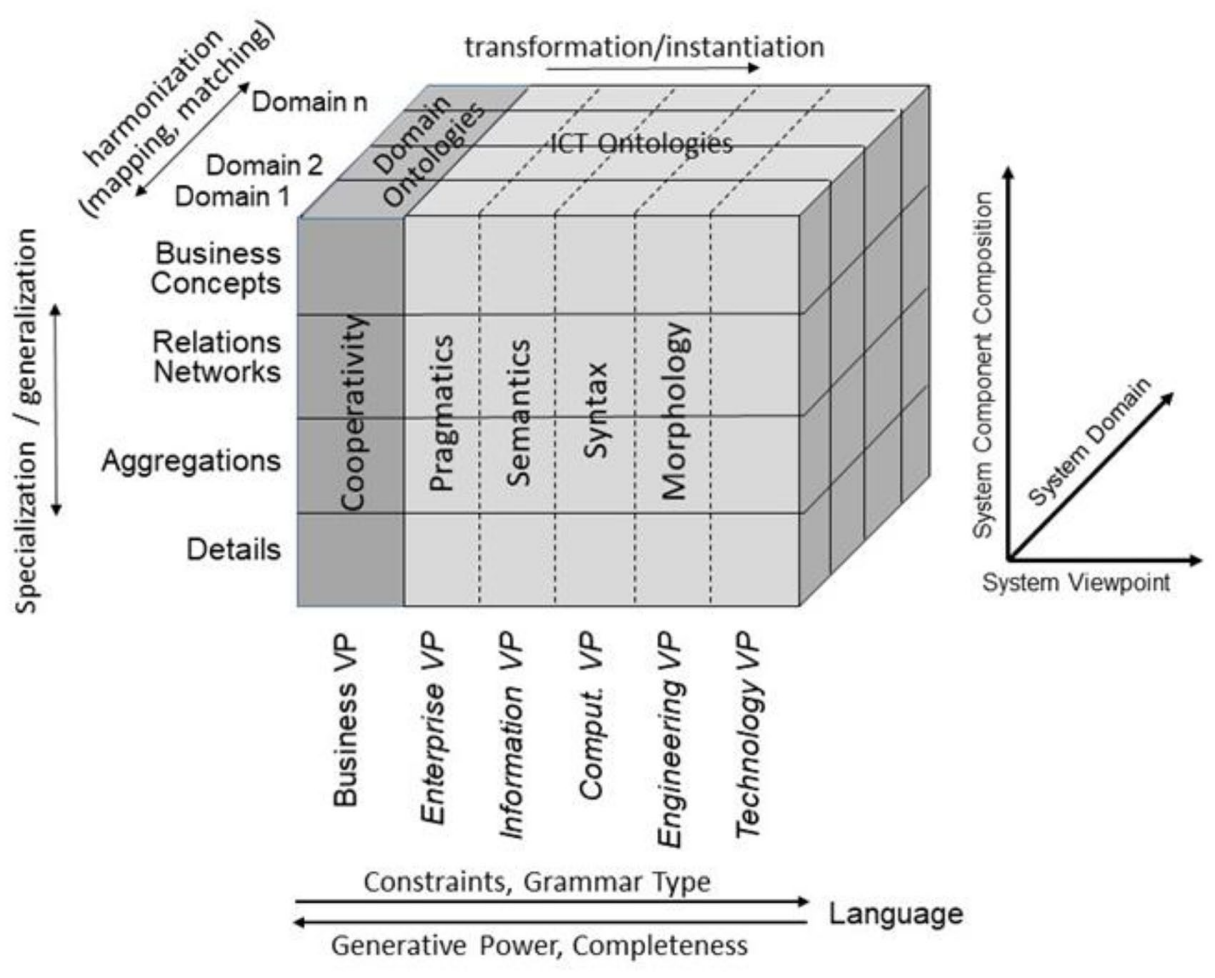
Transformed health and social care ecosystem according to ISO 23903 in a representational focus.

**Figure 2:**
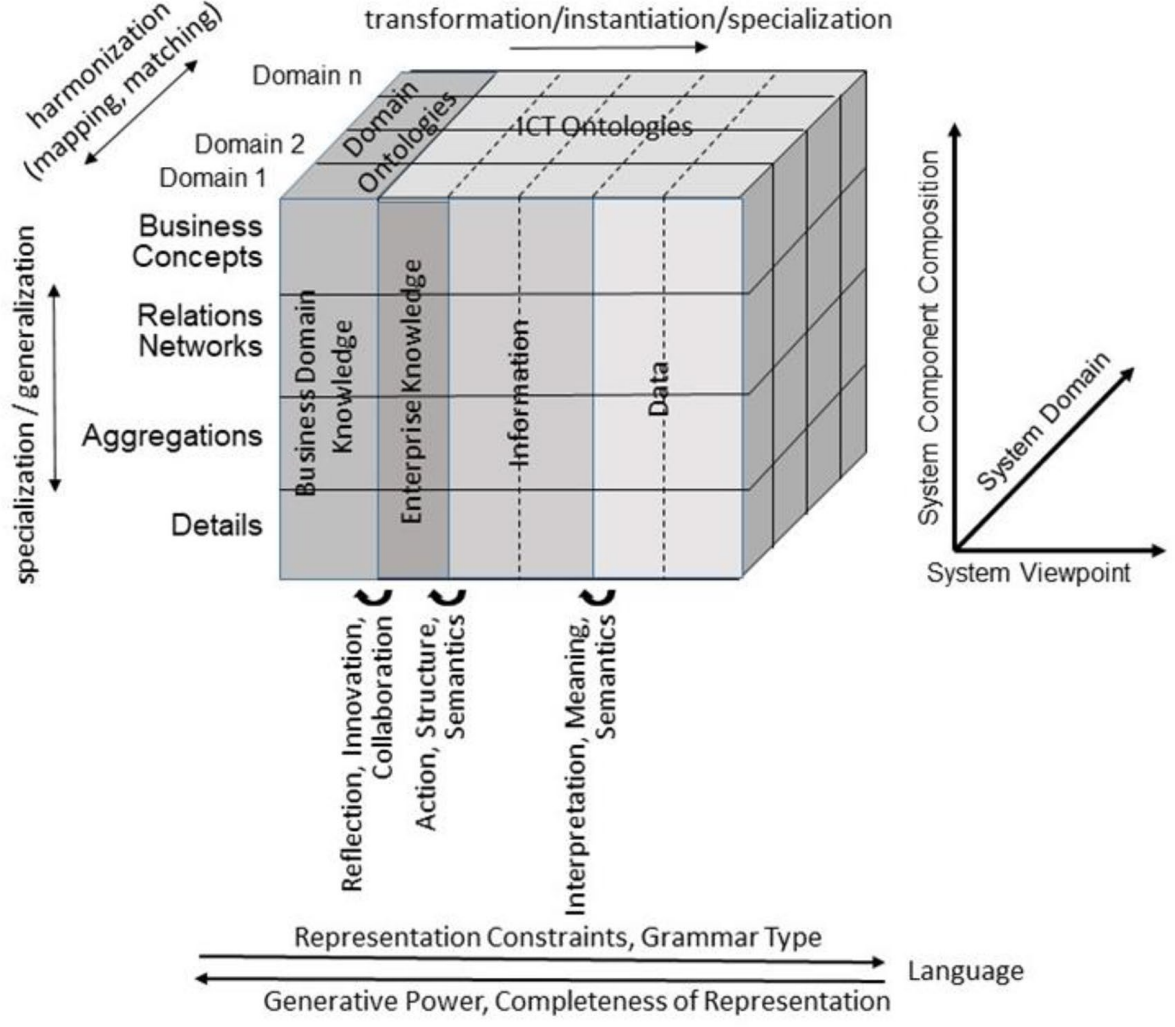
Transformed health and social care ecosystem acc. to ISO 23903 regarding the knowledge types in the ICT solution development process (after (11), changed).

For representing health and social care ecosystems, we have deployed a system-theoretical, architecture-centric, ontology-based, policy-driven approach standardized in ISO 23903:2021 *Interoperability and Integration Reference Architecture – Model and Framework* (5), Figure 1. This standard introduces a top-level architectural model for any multi-domain system, formally representing its components, functions, and interrelations by a cube-shaped model with the three dimensions, *viz*. (a) domains representing specific aspects and perspectives of the system, forming domain-specific sub-systems; (b) generic granularity levels of the system’s elements enabling the composition/decomposition of the system; (c) the viewpoints within its development process. The latter one extends the views defined in ISO/IEC 10746 *Open Distributed Processing – Reference Model* (6–8) *Enterprise, Information, Computational, Engineering and Technology* by the ISO 23903 *Business View*. This view is represented by the domain ontologies harmonized through foundational ontologies (9) aka upper-level ontologies, the different ISO/IEC 10746 views are represented through additional ontologies and specifications of the information technology domain. The former ones include BFO, GFO, UFO, DOLCE, and others, some of them also referred to by the ISO/IEC 21838 Top Level Ontologies (9, 10). The latter ones range from the *Business Process Modeling Language* (BPML) for the Enterprise View through the *Universal Modeling Language* (UML) for the *Information View* and the *Computational View* up to programming languages for the *Engineering View*. As described before, the languages thereby move towards higher expressivity, but more constrained grammars. Capturing knowledge in ontologies enables the understanding of facts and relations by both humans and machines. Thereby, structured and semi-structured knowledge can be represented in different styles at different levels of formalization (2). Figure 2 represents the knowledge types addressed in the ecosystem ICT solution development process. Taking these knowledge types into consideration can provide rigorous design decisions for knowledge representation and reasoning solutions using ontologies (section 6.1. Linguistic opportunities).

## 2. Introduction

A great challenge of 5 PM is to master the tradeoff between (i) the need to constitute clinical cohorts that are large enough for high evidence on the effectiveness of interventions and (ii) the need to account for the individual character of health and disease, which demands personalized decisions for those patients that cannot be considered instances of well-studied large cohorts.

The key to address this problem is data. The more reliable health data are available, the better personalized decisions can be responsibly made on a scientific basis, and the better are data from routine care suited for retrospective investigations. This requires a thorough understanding of (i) what biomedical data are, (ii) which different kinds of data need to be distinguished, and (iii) how data relate to the reality of facts and hypotheses in the domains of biomedical research and personalized healthcare.

We understand by biomedical data all those signals used to support human and machine communication and reasoning about entities (including actors and processes) in the biomedical domain, and which are processed using modeling and programming languages. We have to consider the whole range between structured data (codes, numbers), primarily for machine processing and unstructured data (text, images), mainly for processing by humans.

The way computers deal with data is different from how people do. This raises issues regarding data quality, completeness, processing workflows and interoperability. Data quality is affected not only by measurement inaccuracies, but also by human errors in data handling. Humans also account for the completeness of data collection and registration, but also of the outcome of data retrieval. The fact that the growing amount of biomedical data has far exceeded the limits of human cognition makes automatic data processing indispensable for responsible medical practice. Additionally, different professionals in health care encode data in different ways, using different structures and different languages. This makes data interoperability a major goal which has been largely unfulfilled to date.

Clinical data requires some language to be encoded, with a given vocabulary, syntax, a more or less apparent semantics, embedded into overly diverse and often only implicit pragmatic contexts. This is true for languages used by machines as well as for natural (i.e., human) languages. The following example will demonstrate this.

A hospital laboratory machine plots a set of attributes, values and unit triples (like “Hb; 14; g/dl”) into a tabular structure. Similarly, a clinician inserts codes from a coding system (e.g., ICD-10) into an electronic health record (EHR) together with textual descriptions into a predefined table. In another setting, the clinicians write free-text reports, using the local natural language with its rules and domain-specific terms. In all these cases, not all semantics and contexts are obvious. E.g., the lab machine output does not explain the sampling and analysis techniques. The table with the ICD-10 codes, even correctly filled, may leave open whether the codes refer to diagnostic hypotheses at admission or to clinical evidence at the discharge of a patient. And in the doctor’s report, crucial background information about the patient may be missing because the writer assumes it as known to the reader.

For a long time it has been daily practice for clinicians to supply structured information *via* forms and tables suited to machine processing, e.g., for billing, disease reporting and quality assurance, often redundantly and therefore unwillingly, which explains biases and errors (12). Nevertheless, textual content prevails in EHRs. It is created in various ways. Medical dictation and subsequent transcription by typists play a major role, although text is increasingly entered by medical staff themselves. Spoken language recognition systems are gaining acceptance due to enhancements of trained neural language models (13), which can be adapted to the domain and personalized to their users. No matter how human language is produced, the result is not error-free, particularly when created under time pressure. Several kinds of errors occur, such as typos, grammar violations, other deviations of writing rules such as colloquialisms, ambiguous terms, and undefined short-hand expressions like acronyms are deeply rooted in clinical documentation culture. For a long time, computers had completely failed to reliably extract meaning from this kind of technical language. However, during the last decades, the picture has been changing. The advances in web translation engines like DeepL or Google Translate, and more recently dialogue systems like ChatGPT, have impressively demonstrated how information technology is improving its ability to process human language in a robust manner.

Yet there are largely different flavors of human language as used in the biomedical field. Clinicians use their language and dialect in an *ad-hoc* manner, researchers publish in English, and only the latter one’s texts are aligned with editorial principles before being published. In EHRs, narrative content can be completely unstructured or exhibit several degrees of structure, from document templates up to database tables.

Textual entries come in different degrees of standardization, from a completely unconstrained use of strings of characters over local term collections until shared dictionaries, linked to internationally compatible coding systems like ICD-10 or SNOMED CT. The most sophisticated ones are those that are rooted in some ontological basis, which provides standardized descriptions in logic defining and describing the referents of language entities, i.e., the concrete types of things, e.g., that hepatitis is an inflammation of the liver, that the eye is a sensory organ or that the sigmoid is part of the colon.

In this paper, we will provide an overview of current linguistic and ontological challenges which have to be met to provide a full support of transforming health ecosystems in order to meet precision medicine standards. We will particularly highlight standardization and interoperability aspects regarding formal, controlled representations of clinical and research data, but we will on the other hand consider the users’ point of view. Clinicians require smart support to produce and encode content in a way that machines as well as humans with different backgrounds and contexts can sustainably and reliably understand and process.

The requirement of interoperability and reusability has been formulated for research data by the FAIR (Findable, Accessible, Interoperable, Reusable) data stewardship desiderata (14). We reinforce these principles and advocate their use for all data in the field, which particularly includes routine data in EHRs, a scenario originally not in the focus of FAIR.

To this end, we discuss different formalisms to encode data and knowledge in biomedicine. We hypothesize that precision medicine, requires precision formalisms, such as KR languages with mathematical precision and computable semantics. This desideratum is challenged by a clinical documentation culture, in which narratives are the main carrier of information.

## 3. The perspective of human language

### 3.1. The characteristics of clinical and scholarly language

The crafting of a human language expression regarding its representation of reality principally depends on our innate capability to use a set of symbols and rules. It adjusts to the degree of precision needed by the data exchange use case as well as the background knowledge and thematic scope of the communication partners.

E.g., the expression [i] “MCP, pale, cld, 90/45, 130/min” is precise enough to describe a life-threatening shock situation when uttered by a clinician in an emergency scenario. Clinicians prefer brevity of information-rich messages over redundancy (cf. the following expression [ii]), as long as the recipient of the message can be expected to fill the gaps, here added in italics:

“M*inimally* c*onscious* p*atient with a* pale *face*, cold *skin*, *and an arterial blood pressure measured with a sphygmomanometer on the upper arm resulting in a systolic value of* 90 *mmHg and a diastolic value of* 45 *mmHg, with a pulse rate, measured digitally over a peripheral artery (normally at the wrist), of* 130 beats per minute *on average*.”

The message would even be understood when introducing some noise, such as typing errors and other mistakes like in expression [iii]: “MCP, palle, cld, 90/455, 130/s” (*sic*!).

The correct, unambiguous and precise expression [ii] would, in contrast, not be preferred by the (human) recipient of this information, as perceived wordy and redundant. Similarly, a structured representation (Table 1) would take more time to read than [i]. The shared knowledge of the situational context (in this example the primary assessment of vital signs in an emergency situation), opens a mental map, which already contains the parameters and requires only the values to be added, such as interpreting the frequency value 130/min as heart rate even if the attribute pulse rate’ is not given.

**Table 1:** Tabular representation of the short clinical text “MCP, pale, cold, 90/45, 130/min.”

Emergency case – first assessment	
Consciousness	Minimal
Skin color/face	Pale
Skin temperature	Cold
Systolic arterial pressure (arm) in mmHg	90
Diastolic arterial pressure (arm) in mmHg	45
Pulse rate (beats per minute)	130

In contrast, an automated decision support system would not tolerate any missing parameter, and a wrong unit of measurement could cause considerable harm. The tendency to brevity, the acceptance of noise and the reliance on contextual information to fill gaps and correct errors is characteristic for oral communication, as well as in SMS or social networks posts. Clinical language, equally produced in a hurry, prioritizing content over form, resembles more to the language of WhatsApp messages and tweets than to scholarly publications (15). Table 2 gives an overview of typical characteristics of clinical language.

**Table 2:** Sublanguage characteristics in clinical narratives.

Phenomenon	Example	Elucidation
Telegram style	“left PICA stroke, presented to ED after fall”	Incomplete sentences, sketchy style
Colloquialisms	“pothole sign”, “snorkel”	Milieu-specific sub-languages
*Ad-hoc* abbreviations	“infiltr”	Truncation (“infiltrated mucosa”)
Ambiguous short forms	“RTA”	“Road traffic accident”, “Renal-tubular acidosis”
Short forms of regional or local scope	“LDS Hospital” “St. p.”	“Latter-Day-Saints Hospital” “Status post” = “History of”
Conventionalized Latin abbreviations	“V mors can dig V dext”	“Vulnus morsum canis digiti quinti dextri” = “dig bite in the right 5th finger” (common in some European languages)
Spelling errors, typos	“Astra-Seneca,” “Hipotireose”	accidental or systematic (e.g., 2nd language speakers)
Spelling variants	“Esophagus”, “Oesophagus”	e.g. American vs. British English
Single noun compounds	“Ibuprofenintoxikation”	Non-lexicalized long words (in languages such as German, Swedish)
Anaphora	“adenoCa rect pN + MX G2 (…). tumor excised *in toto*” “no blood in stomach (…). mult mucosal erosions”	Understanding requires reference to surrounding text, “Tumor” coreferential to adenocarcinoma described in left context “mucosal erosions” refined to “erosions of gastric mucosa”
Negations	“No evidence of pneumonia” “Pulmones: nihil,” “metastasenfrei”	non-standard, jargon-like
Epistemic (uncertain, speculative) contexts	“susp MI, DD lung embolism”	suspected diagnosis, differential diagnosis
Temporal contexts	“h/o Covid-19” “Streptokokkenangina 06/16”	“history of” coarse grained dates (mm/yy)
Other contexts	“father: pancreas ca” “refrained from resuscitation”	family history plans not executed

Published texts, in contrast, are carefully copy-edited and follow guidelines, which, e.g., prevent the use of unorthodox spelling or undefined acronyms. The reader of a scientific paper would not be left in the dark, whether “MCP” means “Monocalcium Phosphate”, “Metacarpophalangeal,” “Medical College of Pennsylvania” or, like in our example, “Minimally conscious patient”.

The observation that clinical narratives are often characterized by complete freedom in text design, forms a contrast with the enormous amount of effort invested in vocabulary normalization over decades (16). To name just a few, ICD-10 (17) is a worldwide standard for encoding medical conditions. Phenotype data can be coded by MedDRA (18) or the Human Phenotype Ontology (19), LOINC (20) is used as a controlled vocabulary for laboratory and other observational characteristics, ATC (21) and RXNorm (22) describe drugs and drug products, and SNOMED CT (23), an ontology-based terminology, claims to provide codes for the whole range of EHR content. For scholarly publications, the controlled MeSH vocabulary (24) is used for abstracting the key topics a scientific paper is about. Medical terminology systems are heterogeneous and overlapping. The UMLS (Unified Medical Language System) (25), maintained by the US National Library of Medicine, is a long-lasting effort of the biomedical informatics community to collect and to map medical terms from over a hundred terminology systems, thus facilitating interoperation, biomedical language processing and retrieval.

Although clinical terminology systems are often referred to by the term “controlled vocabulary” (CV), this does not imply that they play a significant role for controlling the terms used when producing clinical or scholarly narratives. Their main purposes are the support of structured data entry into forms such as for health statistics, quality assurance, reporting, and billing, the semantic annotation of article content in literature databases and the standardization of clinical data sets for research, e.g., within the Medical Outcomes Partnership (OMOP) Common Data Model (26).

Most terminology systems are primarily models of human language. They organize words and cohesive multiword sequences, normally referred to as “domain terms” or “terminological units.” These units are connected by semantic relations such as synonymy and hyponymy. From a class of domain terms, normally one, typically self-explaining term is flagged as the preferred term. Its meaning is further explained by textual elucidations. Semantic relations in informal terminology systems, however, rely more on context-dependent human judgment than on crisp, objective criteria. E.g., the fact that “Animal” is a hypernym of “Human” may be trivial for a biologist, but debatable for a jurist. For a chemist “alcohol” is clearly a hypernym of “ethanol,” whereas a general practitioner uses them as synonyms. The meaning of “fear,” “anxiety” and “worry” has no clear boundaries, so that whether they are considered synonyms is much dependent on individual judgment and situational context.

### 3.2. The processing of biomedical language by computers

For decades, natural language processing (NLP) has been seen as an important and relevant application area of artificial intelligence, particularly because it bears the promise to bridge between humans and machines. Only in the last decade, however, NLP technology has reached enough maturity to play an ever-increasing role in application software, which determines ever larger parts of our everyday life, particularly in mobile applications.

When applying NLP technology to clinical narratives or scholarly publications, the main focus is on text mining, by use of different information extraction (IE) methods (27). IE systems analyze text structure and content in order to fill pre-structured information templates. An example is the processing of a pathology report in order to populate records of a tumor registry (28). This task of distilling structured data from unstructured text serves many purposes. Applied to clinical text, structured extracts can be used for all the documentation and annotation purposes as addressed in the previous section.

The complexity of the extracted information ranges from simple binary variables such as *Smoker* (yes/no), to parameters with numerical values for a parameter like *Oxygen Saturation* (e.g., 98%) to codes from a terminology system with up to hundreds of thousands of possible values. Their standardized meaning, is then often further contextualized by information models such as HL7-FHIR (29, 30), which provide information templates that represent the context in which the codes have to be interpreted, e.g., the role a disease code plays within a diagnostic expression. Instantiated FHIR resources specify, e.g., the time of a diagnosis and whether it refers to a current health problem, a resolved one, one in the patient’s family, or a hypothesis raised by a clinician.

Text mining analyzes and normalizes linguistic units of different granularity. The largest unit is the document. Documents can be distinguished by types (e.g., discharge summary, radiology report, progress note) as well as subdivided into sections. Sections can also be assigned a type, e.g., *Diagnosis*, *Evolution*, *Laboratory*, *Medication* etc. in clinical documents or *Introduction*, *Methods*, *Results* etc. in scholarly publications. Within sections, sentence-spanning phenomena such as anaphors (see Table 2) or semantic relations need to be identified for a complete understanding, e.g., to link the mention of a procedure with the mention of an anatomical structure. Sentences are decomposed into smaller units (chunks) using shallow parsing, supported by the analysis of parts of speech (POS), i.e., the identification of word classes such as *Noun*, *Verb*, *Adjective* etc. Within chunks, text passages that can be mapped to a controlled term are finally identified by matching against a domain vocabulary, such as constituted by or linked to one of the mentioned terminology systems. Such passages can be short (e.g., “cough”), but also complex, such as “non-intensive COVID-19 infection with positive vaccination status.” At this level, two NLP tasks must be distinguished. First, the identification and delineation of a text passage to which a specific semantic type like *Disease*, *Symptom*, *Medication*, *Proper Name*, *Institution* can be ascribed. This is known as *Named Entity Recognition*. Second, the mapping of the identified candidate to the target vocabulary, which is known as *Named Entity Normalization, Entity Linking* or *Concept Mapping* (31–33). Pioneering systems for the English medical language are cTAKES (34), MetaMap (35) and MedKAT/P (36). In these systems, text content is automatically matched against terms in terminology systems and tagged with their codes.

Many use cases require connecting text passages, after normalization, to a temporal context. Clinical texts often do not report on facts chronologically, and time differences between important events of a patient history, e.g., between the first diagnosis of a tumor and its recurrence after therapy, are of prime interest. Standards like TimeML (37) have been proposed, as well as algorithms for the identification of time events, e.g., HeidelTime (38). The evaluation of temporal relations and putting events into context has also been addressed by the 2012 i2b2 Challenge on evaluating temporal relations in clinical text (39). Equally important is the identification of the negation context of a text passage, where NegEx (40, 41) has been optimized to different domains and languages (42–44), but the generalizability of the approaches is still missing (45).

The described analysis steps are implemented in classical text mining systems as software known as automated annotators or taggers. A common framework is Apache UIMA (46). Individual text analysis modules communicate in a processing pipeline by enriching a complex data structure, which incrementally adds information to the text under scrutiny. This information is represented by typed, access-optimized feature structures, which assign types and properties to a span of characters corresponding to text passages. Component repositories like DKPro (47) based on uimaFIT (48) support a flexible composition of use case specific building blocks for NLP systems, which implement specific functionalities in the chain. Important Python based NLP frameworks which have to be mentioned in this scope are spaCy (49) and Spark NLP (50).

A major paradigm shift has occurred in NLP during the last decade, driven by the unprecedented rise of artificial neural networks (ANNs) for machine learning, known as Deep Learning. Although the principles of ANNs have been formulated about 80 years ago, only now their combination with powerful computer architectures, big amounts of data, and innovative algorithms has unveiled their potential. Their popularity has been supported by practical program libraries such as KERAS, TensorFlow, PyTorch, and HuggingFace (51–53).

Deep learning approaches have largely replaced “shallow” machine learning methods in recent years, especially because features that are productive for learning success no longer require time-consuming feature engineering (54). Revolutionary for natural language processing are embeddings, which are computed semantic representations of text passages in vector spaces of medium dimension (e.g., 300), learnt from textual data. The embedding-based vector representation can be used for example to identify related term-candidates by applying different distant metrics like the cosine-similarity within this n-dimensional space. More recent architectures, particularly BERT (55) and GPT (56) can provide so-called contextualized embeddings (57), in contrast to first non-contextualized approaches like Word2Vec (58), GloVe (59) and fastText (60). For a given linguistic unit, these can incorporate the relevance of preceding units to their vector representation and thus distinguish between homonyms (e.g., “delivery” in “drug delivery mechanisms” from “normal labor and delivery”). For many of the model-based extraction tasks, Deep Learning, specifically the use of transformer-based architectures is now standard, and in some cases specific tasks no longer rely on the interaction of pipeline elements, but can be handled with an independently trained model, also known as end-to-end processing.

Training neural networks with sufficiently large amounts of data is “expensive” in terms of hardware requirements and processing time. This is specifically true when it comes to the generation of language models, which are usually downstreamed in a second step to a specific problem domain like named entity recognition or document classification, often referred to as transfer learning (61). Just some openly available language models exist for the clinical domain (62) which can be leveraged adequately for this kind of model-based problem adaption.

Nevertheless, traditional, “low-tech” rule-based approaches still find their application, particularly where scarceness of training data meets in-depth expert knowledge about the domain. Training models only on publicly available data is by far not enough to reach a good quality, particularly when it comes to entity normalization and disambiguation, and even more for languages other than English (63). This is particularly important for clinical narratives, because contrary to scholarly publications, most clinical texts, from a global perspective, are written in languages other than English.

It should not be forgotten that speech recognition technology, nowadays mostly implemented as recurrent neural networks like LSTMs (13) is becoming increasingly popular. A randomized study from 2015 showed an increase in productivity by physicians using this technology (64). In the meantime, speech recognition software has been shown to be faster and more accurate than typing, but the acceptance by clinicians still leaves a lot to be desired (65).

## 4. Ontological perspective

### 4.1. The need for semantic integration of health data

For the most part, medical research and medical standards of care are driven by international scientific and professional communities. Researchers and practitioners with different linguistic and cultural backgrounds have little problems when discussing medical issues, as long as a certain level of command of English and the knowledge of English medical terminology is guaranteed. Thus, experts from Baltimore, Bamako, Beijing, Berlin, Bogotá and Brisbane can get together to discuss issues of state-of-the-art clinical diagnosis and therapy. The picture changes already when we want to automatically integrate data from two hospitals in the same city: All kinds of major problems will arise, and harmonization of data from their respective EHRs requires human labor to an often-prohibitive extent. Similar challenges arise whenever we attempt to bridge between EHR data and content of scholarly publications and databases.

The main desideratum is semantic interoperability. The role ontologies and other semantic standards can play in fostering or creating semantic interoperability of heterogeneous clinical and scholarly data to fulfill the pHealth requirements will be discussed in the following section.

### 4.2. Ontologies as a special type of terminology systems

Ontologies have been important resources in computer science for decades, accompanied by a variety of tools and representational languages. Unfortunately, the way how they were conceived and defined, as well as the purposes for which they have been built, has shown great variation. We have introduced the notion of a terminology system in the previous section, and often the term “ontology” is also used to refer to them. We consider this view to be of little use. Instead, we introduce the clear bipartition, highlighting “ontologies” as “formal ontologies” (66–69), contrasting them with the large number of terminology systems that are not based on formal semantics, such as ICD, ICF, MeSH, MedDRA, but also the UMLS Metathesaurus.

Formal ontologies are “precise mathematical formulations” (70), or more concretely, logic-based definitions and elucidations of the types of entities of a domain and the way they are related. This requires a computer-interpretable language, which typically distinguishes between individuals, classes and properties. The main purposes of ontologies are (i) to support knowledge representation and reasoning and (ii) to foster interoperability by standardized descriptions. One example is the automatic assignment of a class to individuals based on a computer-interpretable, axiomatic specification of the inclusion criteria for the class. In Utecht et al. (71) demonstrated that an ontology-based system can categorize potential drug–drug interaction (PDDI) evidence items into different types of evidence items based on the answers to a small set of questions. They tested RDF/OWL data representing such questions for 30 evidence items and showed that automatic inference was able to determine the proper evidence type category from a list of approximately 40 categories based on this small number of simpler questions. This is a proof-of-concept for a decision support infrastructure that frees the evidence evaluator from mastering relatively complex written evidence type definitions and allows for ontology-driven decision support.

The fact that natural language plays only a secondary role in formal ontologies is not a contradiction to what we wrote in the previous sections. The main difference is that the types of entities characteristic for a domain is the starting point when building an ontology, and not the meaning of domain terms in a particular natural language in the first place. In no way this should detract from the importance of domain language dictionaries. But the concerns are strictly divided: the ontological standardization of domain entities on the one hand, and on the other hand the anchoring of domain terminologies in several natural languages and dialects. This means, in practice, that synonyms and term variants in different languages are then linked to ontology IDs. At least one preferred term, often referred to as “label” is needed in order to make the ontology understandable by humans. Enriching it by additional terms is often done by the ontology builders themselves. Here, the ontology also fulfills the role of a dictionary.

Standardization has been an important issue regarding the formal languages employed by ontologies. Based on description logics (72), promoted by the W3C, the declarative Ontology Web Language OWL has become widely accepted. OWL is devised to verify the consistency of a set of logic-based axioms from which implicit knowledge can be made explicit by so-called description logics reasoners (73).

Equally, by the W3C, the Simple Knowledge Organization System SKOS (74) has been promoted as a representation of systems that informally structure a domain by its terminology. SKOS’ main objective is to enable easy publication and use of vocabularies as linked data. Both OWL and SKOS are part of the Semantic Web family of standards built upon RDF and RDFS, both are used with data represented in the universal Resource Description Framework (RDF) of the Semantic web (75, 76). The abstract syntax of RDF – which does not enforce any strict semantic interpretation, has at its center the representation of data as triples, i.e., statements consisting of subject, predicate and object (75). The simple, very small structure can be linked together using International Resource Identifiers (IRIs) for each entity in the domain of discourse (75), thus enabling the creation of complex knowledge graphs.

The following example may illustrate the difference between the two languages. In SKOS, the triple < “Homo sapiens”; skos:broader; “Living organism” > expresses that the meaning of the expression “Living organism” is conceived as broader than the expression “Homo sapiens.” In OWL, the triple < “Homo sapiens”; owl:subclassOf; “Living organism” > has the status of an axiom. It means that the class of all individuals of the type “Homo sapiens” is included in the class of all individuals of the type “Living organism.” Whereas in SKOS, the relata are human language expressions like words and terms, in OWL “Homo sapiens” and “Living organism” are no more than human-readable class labels that make the ontology human-readable. The exact meaning of the classes requires further definitions of these types. According to these definitions, the axiom could be questioned by the fact that the class labeled as “Homo sapiens,” according to how it is defined, may also include dead persons. As SKOS has no formal semantics, it is at the discretion of the users to approve the statement < “Homo sapiens”; skos:broader; “Living organism” > as largely appropriate, despite boundary cases like the abovementioned one.

Thus, RDF-based Semantic Web standards constitute a framework that equally accounts for ontologies as carefully constructed cornerstones, for informal knowledge organization systems as bridges to human language, and the breadth of knowledge representation built upon it.

### 4.3. Standardization aspect of ontologies

Whereas computer science has always had a functional look on ontologies – an ontology is as good as it supports a given use case and its particular world view, life sciences have put much more emphasis on the interoperability aspect of ontologies. For instance, the Open Biological and Biomedical (OBO) Foundry (77) established a set of principles (78), ontologies have to comply with: orthogonality, open access, instantiated in a language that allows computer-interpretability, and use of common, shared identifiers (79). Over the last years, the OBO Foundry community has worked to improve those principles and increase compliance for the OBO Foundry to become a key resource towards making biomedical data Findable, Accessible, Interoperable, and Reusable (FAIR) (80).

Orthogonality means that each ontology has its scope limited to entities of clearly defined types and scopes. It points to a framework of shared fundamental categories: chemical entities and roles such as in ChEBI, anatomical entities in the FMA, cell components, biological processes in the biological process and molecular “function” (activity) in the Gene ontology. Other examples are qualities in the human phenotype ontologies, locations in the environment ontology. All this points to high-level types of a common “upper level” (9), which is the focus of interest of the Foundational Ontology/Applied Ontology community. Foundational upper-level types, properties and related axioms (e.g., that a process is located in some space or that an immaterial entity cannot have material entities as parts) strongly constrain the modeling freedom of the ontology engineer, for the benefit of interoperability.

**Figure 3:**
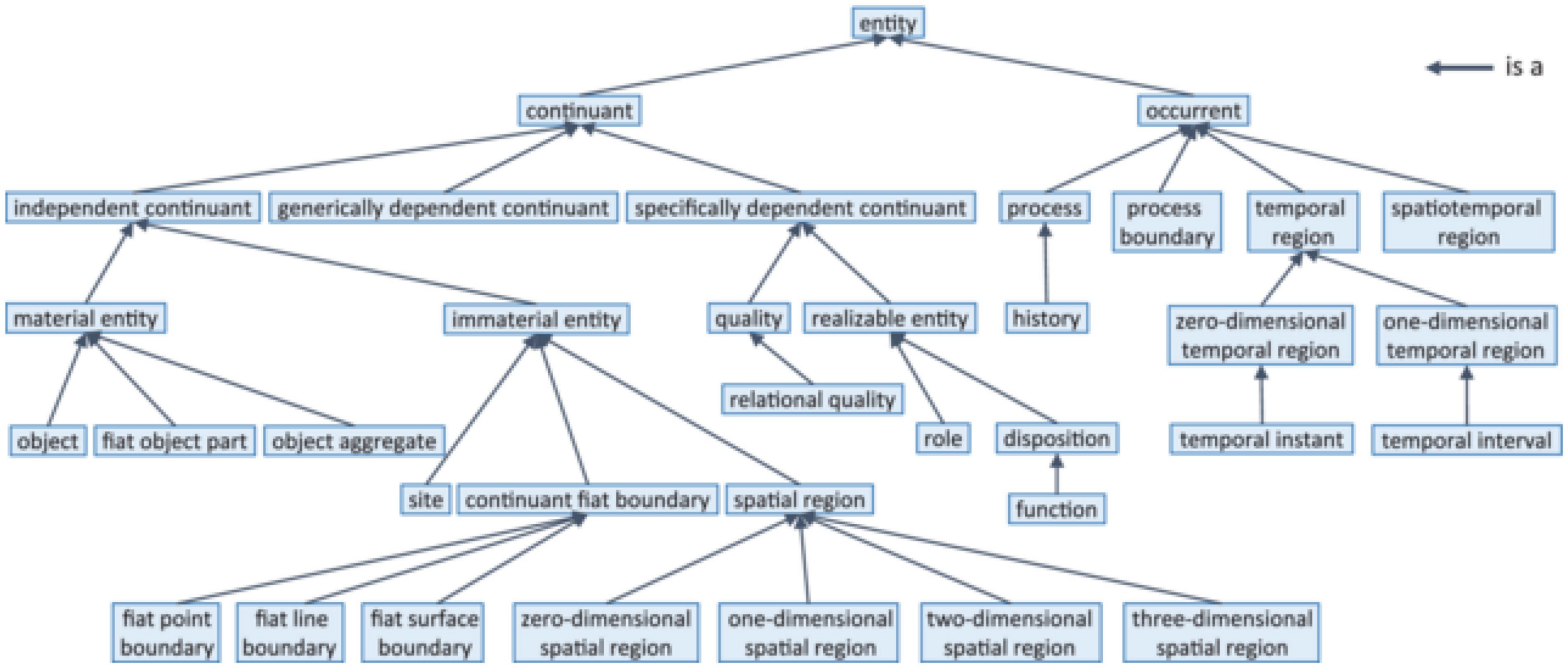
BFO-2020 taxonomy (according to ISO 21838-2 (10)).

In the domain of life science, BFO (81, 82), the Basic Formal Ontology has found the widest acceptance. Figure 3 demonstrates its upper level.

The fact that BFO 2020 has become an ISO standard (10) sheds light on a new view on ontologies, namely the standardization (83) of entities in the field of science. In engineering it has always been obvious that a narrative description of an artifact would not be sufficient for producing interoperable industry-standard products. Only exact technical specifications guarantee the smooth interaction of technical components like plugs and sockets. The argument in favor of biomedical ontologies is that like bits and pieces of industrial artifacts require adherence to mathematically precise standards to be exchangeable and interoperable, entities of interest for precision-oriented science and health care require the same accuracy in terms of ontological definition and delineation. Such entities of interest range from biomolecules and pathways over body parts, disease processes, quantities and qualities, pathogens, medical devices up to all kinds of interventions and complex business processes in 5 PM contexts.

Apart from BFO, standardization has also been an issue in biomedical terminologies, particular in the case of SNOMED CT (84), which set off as an international terminology for EHRs, but which then increasingly adopted principles of formal ontology and logic, so that it can now be seen as clinical ontology of high coverage and granularity.

### 4.4. Assessing biomedical ontologies

However, not all healthcare and life sciences ontology developers share the view that ontologies should be interoperability standards. Up until now, numerous project-specific ontologies have been built without any interoperability or standardization interest. They are maintained for the duration of a certain project and are then abandoned. They do not refer to foundational ontologies, nor do they re-use content from other domain ontologies. Such resources amount to many hundreds, which can be inspected *via* BioPortal (85), a collection of ontologies and ontology-like representations, regardless of their formal rigor and maintenance status.

A critical analysis is therefore appropriate. When reviewing the ontologies created in recent years, we see, on the one hand, increasing acceptance of good practice design principles, at least where there are enough resources for ontology curation, such as in SNOMED CT and some of the OBO Foundry ontologies. On the other hand, despite all research and education in the field of Applied Ontology, numerous ontologies continue being constructed idiosyncratically, for specific use cases only, and without concern for interoperability. Such ontologies often ignore the strict requirements of logic, do not use machine reasoning and do not subscribe to any upper-level ontology. They often contain workarounds with the purpose to represent what ontologies are not meant to express, namely fuzzy, context-dependent or probabilistic representations. The fact that the use of the logic of OWL is restricted to axioms that are universally true, is often not taken into account in all its consequences. Even people with sufficient training in ontology are not aware of the fundamental differences between the statement “tobacco causes lung cancer” and the statement “tobacco contains nicotine.” Only the latter one can be properly expressed by OWL, because tobacco always contains nicotine. The former one, in contrast, makes a probabilistic statement about populations, regarding a non-accidental co-occurrence between smokers and people with lung cancer, which does not preclude smokers without cancer and lung cancer patients that never smoked.

Another pitfall is improper or ambiguous labeling. Bioportal currently displays 58 ontologies with a class labeled “heart,” although the heart of an adult fly, a mouse embryo or a human heart transplant do not have much in common. Absurd mappings derive from the matching of labels, e.g., of “cold” to “chronic obstructive lung disease” (for which the acronym “COLD” is used). Even good ontologies often do not have a good labeling discipline, because the language expressions used as labels have different meanings in different communities. In other cases, ontologies like the Gene Ontology (GO) do not have more than one label per class, which leads to the practice to refer to GO classes as “GO terms,” which is confusing for anybody with a terminology of a linguistics background.

Finally, a complicating factor when constructing ontologies is the continuous nature of many natural kinds (86). Instances of hearts, brains and muscles – be it from mice, humans or flies – do not have sharp boundaries that delineate them from the neighboring anatomical structures, such as an engine in a vehicle. Heart surgeons would consider the pericardial sac and parts of the great vessels as part of the heart – as they transplant it together with the heart proper – opposed to anatomists, who share an ontogenetic (embryological) perspective. All these subtleties are seldomly made explicit in ontologies, so that the risk that re-using ontological content created in a different context produces unwanted effects, is considerable. After all, considering those human-made and therefore imperfect ontologies comes back to the problem we have with natural language as a means to encode information and knowledge, viz. the need to consider context and the acceptance of having fuzzy, partly conflicting, partly ambiguous representations.

## 5. Integration of ontologies and natural language for 5 PM

We now move to discuss the interface between ontologies and natural language technologies, with the goal to support formal, unambiguous and canonical representations of structured and unstructured data in the field of 5 PM, encompassing research data as well as real-world data from EHRs.

### 5.1. Canonic representations of narrative content

It is unrealistic that non-standardized and low-structured narrative data, currently prevailing in EHRs (87, 88) will be replaced at some point by completely structured and standardized documentation, as little as it is likely that future scholars will publish all their data according to the FAIR criteria. Human language will probably never lose its function in clinical and scholarly communication and documentation, due to its capability to describe facts and events in a flexible and granular way, just to the degree that it is understood by clinicians or researchers that share the same or similar contexts. The challenge is therefore that heterogeneous data of all kinds is analyzed and semantically interpreted in a way that leads to maximally standardized and interoperable representations. This is why semantic standards with a big, international user community, e.g., SNOMED CT, LOINC, and FHIR should be preferred as target representations in biomedical data normalization workflows.

### 5.2. The resource problem

The performance of NLP systems depends crucially on the available resources. These include terminology systems and corpora, as well as language models derived from the latter. For the clinical language, there is a great need to catch up here, due to the lack of models tailored to clinical language, as well as natural languages other than English (62). So does the UMLS aggregate an impressive variety of terminology systems, but mainly in English, thus limiting terminology support for other languages. SNOMED CT has been translated and is being maintained in several languages, incurring high costs and efforts, and results lag behind, particularly for smaller languages. In addition, the existence of a translation does not necessarily mean that it is suitable for NLP applications. Terminology systems tend to be normative in nature, so that terms are ideally unambiguous and self-explanatory. This often does not reflect clinical language use. E.g., in a corpus of 30,000 cardiology physician letters from an Austrian hospital (89), the authors did not find the word “Elektrokardiogram” a single time – contrasting with thousands of occurrences of the acronym “EKG.” For “liver metastases,” the term “sekundär malign levertumör” (secondary malign levertumör) is found in the overall Swedish translation of SNOMED CT, for which not a single use can be found in the entire web, while the clinically common “levermetastaser” has over 200,000 Google hits, but is missing in the Swedish SNOMED version. The EU project ASSESS-CT (90) propagated the creation of so-called interface terminologies, collections of technical terms that primarily represent the language used in the clinic. Examples for interface terminologies are the German ICD-10 alphabet (91) or the Austrian interface terminology for SNOMED CT (92). Corpora, i.e., text collections are essential for training NLP systems as well as for their evaluation, for example in shared tasks, which are scientific competitions like i2b2/n2c2 or the ShARe/CLEF eHealth and SemEval challenges (93), partly re-using narratives from the most prominent clinical language resource MIMIC (94). The more such open resources exist for a language group, the better synergies can be exploited by developers of NLP systems. For example, a large part of the quality of the Google translator is due to the simple fact that Google has direct access to gigantic amounts of multilingual texts.

Clinical researchers can only dream of this. Clinical content is highly confidential, so that only reliably anonymized data can be considered for the training of NLP systems. Anonymization means marking names of persons and institutions (95) in order to remove the direct reference to persons and institutions, an absolute requirement for clinical texts to be processed for purposes other than patient care and by persons other than those directly caring. What content is relevant for de-identification – PHI (protected health information) – is often not specified, so that many countries apply the HIPAA safe harbor criteria (96), developed in the USA. However, concerns regarding the release of anonymized clinical text samples are still enormous. As an example, the completely manually anonymized German-language annotated clinical corpus BRONCO could only be released after it was divided into randomly arranged individual sentences, the coherence of which could demonstrably not be restored (97). Other ways of providing open clinical corpora include the creation of completely synthetic texts using machine learning (98). Access in a controlled setting for the use case specific adaptation of clinical NLP systems is indispensable for any reasonable use of narrative data in combination with enhanced standardized and interoperable clinical phenotype representations into a transformed health ecosystem.

### 5.3. Manual annotation as a fundamental task

Annotated corpora, i.e., text collections that were manually enriched by labels that describe the text according to its syntactic and semantic features, is not only an enormously resource-intensive effort, but also of utmost importance for entity normalization and semantic relation detection, but also text classification, sentiment analysis, question-answering and other tasks. Models trained with annotated corpora enable machines to understand the meaning of language in clinical narratives, and allow for more accurate analysis of medical data, particularly in P5 medicine settings.

Semantic annotations should be guided by the same interoperability resources and using the same interoperability standards as expected for the target representation of clinical content. Only under these circumstances, consistency in annotation can be reached, and principled annotation guidelines can be formulated (99). Such annotation guidelines have to bridge between shared representations of the portion of reality the texts are about (health care scenarios for EHR content, lab procedures, clinical research paradigms, scientific methodology and argument when it is about scholarly content) on the one hand, and the text surface on the other hand. This means to link text passages to the ontology classes they denote (or to abstractions thereof, such as upper-level categories like *Body Part*), which requires a deep knowledge of the underlying ontology as well as familiarity with the domain, particularly in the case of ambiguous text passages such as acronyms. Entity normalization, i.e., the linkage of words and text passages to ontology identifiers such as SNOMED CT or LOINC codes is only the first step. Equally important is their linkage to contextual or temporal modifiers, in order to represent the entirety of a statement, e.g., whether a diagnosis is confirmed, suspected or negated, when an examination was done or when a recurrence of a disease occurred. Finally, annotations often need to be linked by relations, such as procedures or observations with the related anatomical sites, operations with devices or infections with pathogens, but also sequences of events by their temporal order and possibly causation. The relations used for annotation should be consistent with the underlying standards, such as *Finding site* in SNOMED CT or *verificationStatus* in FHIR. For assessing the quality of the manual annotations, the inter-rater agreement between annotators is very often measured *via* Cohen’s kappa, Fleiss kappa or F1 value (100, 101). The higher the value, the higher the agreement of two human annotators for a specific annotation task. An excellent overview of manual annotation tools is given by Neves and Ševa (102).

### 5.4. Pervasiveness of semantic technology in health informatics

This section is primarily concerned with the interplay between health standards, terminology systems like classifications and terminologies, as well as semantic web technologies. This is an important aspect of health data infrastructures and, in order to understand ontology-related challenges to creating transformed health ecosystems, these categories need to be considered.

At this time, there is wide-spread agreement that semantic data harmonization and integration are necessary to move forward biomedical research on a number of key areas in the field (103–106). The recent COVID-19 pandemic showcased the importance of fast and reliable data and knowledge management to support COVID-19 (107, 108) research. This research is crucial to reign in the spread of COVID-19 and effectively improve patient outcomes. While this trend is certainly welcome and hopefully is the first step into deeper involvement of the biomedical informatics community in research regarding semantic technology, there is still much work to do in order to reach the pervasiveness of semantic technology in health informatics for the benefit of P5 medicine.

In a recent systematic literature review on semantic interoperability in health record standards (104), de Mello et al. proposed a five-category taxonomy for research in that area: (1) Health standards (e.g., OpenEHR, HL7, DICOM), (2) Classification and Terminologies (e.g., ICD, LOINC, SNOMED CT, MeSH), (3) Semantic Web (e.g., OWL, RDF, SPARQL, SKOS), (4) Storage (e.g., Multi-model, Semantic Web based, graph database), (5) Evaluation (e.g., Usability, Functional test) (104). The authors of the review concede that many of the research papers included fit in more than one category, which means that the classes in this taxonomy are clearly not mutually exclusive (104). For instance, both the ontology SNOMED CT and the language OWL are considered standards. The review also found that the use of ontologies and other Semantic Web technologies (SWTs) is motivated by the possibility to create logical inferences and rules from them. This finding leads to the core of the difference between an SWT-based approach to semantic data integration and harmonization and the use of the other categories (104).

The fact that biomedical researchers chose SWTs due to the ability to create new data points or run more inclusive queries using logical inference is a highly relevant point. One example for such an inference used in querying data is if a biobank stores a specimen labeled “cerebellum” and the biobank uses an anatomy ontology to specify that every cerebellum is a part of some brain, a biobank user can retrieve that specimen when running a query over all specimens of the brain or its parts, rather than running multiple strings (brain, cerebellum etc., possibly also including all synonyms) in one or multiple queries. The fact that the cerebellum is part of the brain is explicitly stated as part of the knowledge system and not externalized in the mind of some database employees. An example for a new data point created based on automatic inference is if a study has the information that the pediatric patient Jane Doe lives with their parent John Doe and the system also has the information that John Doe is a smoker, a knowledge management system containing a computer-interpretable definition of a smoking household as a household that has at least one smoker as a member, the system automatically infer that Jane Doe is living in a smoking household. Beyond these use cases, the use of ontologies and other SWTs also allows automatic sorting of individual entities in categories that have computer-interpretable definitions, as (71) demonstrate.

One application of SWT is Knowledge Representation and Reasoning (KRR) (109), a core area of Artificial Intelligence (110, 111). KRR provides the basis for “representing, maintaining, and manipulating knowledge about an application domain” (111), such as medicine. According to Lakemeyer and Nebel (111) the core elements to meet that aim are explicitness and declarativeness. Explicitness means that the knowledge needs to be stored in a knowledge base along with formal representations describing it in an unambiguous way, and declarativeness means the “meaning of the representation can be specified without reference to how the knowledge is applied procedurally, implying some sort of logical methodology behind it” (111). It is clear that this specification of knowledge representation is largely about the way that the meaning, or semantics, of the knowledge is specified. It is implicit in the Lakemeyer’s and Nebel’s specification that the call for explicitness entails the requirement for the knowledge to be represented in a computer-interpretable language. The motivation to add reasoning as a crucial component to knowledge representation is, according to Brachman and Levesque (110), that this allows to infer new, often actionable knowledge, such as a potential adverse reaction to a drug inferred from previous drug reactions. In sum, when talking about the area of KRR, the semantics of knowledge rests on the formalization of the knowledge in a computer-interpretable language, the coding of unambiguous representation of its meaning in a way that does not refer to its operationalization. Keeping this in mind, highlights a number of obvious gaps in how the biomedical informatics community uses the term “semantics.”

In 2018, Brochhausen et al. pointed out, that there might be a disconnect regarding the use of the terms “semantic” or “semantics” in biomedical informatics: terminology systems, often in combination with Common Data Models (CDMs) are implemented to provide semantics and foster semantic integration and researchers using those resources and claim their data is semantically integrated and computer-interpretable (112). None of the systems assessed in their study exhibited any features that amount to semantics, in the sense of the KRR community. Most of the tools featured human-interpretable definitions. To help address that communicative gap, Brochhausen et al. proposed the term computable semantics (112), which basically applies to the specification of semantics in a KRR context. They proposed to use a sorting task to assess whether a computer understands the data as a low hurdle measure to test for the existence of semantics.

But if this communicative gap exists, what is meant when biomedical informatics papers talk about semantics or semantic interoperability. The multitude of interpretations of the terms “semantic,” “semantics,” or “semantic interoperability” warrants a systematic review, which is out of scope for this paper. However, we want to highlight some possible interpretations and how they compare or relate to the SWT approach using ontologies.

One traditional perspective is that achieving semantic interoperability relies on and can be achieved by the use of standardized terminologies or controlled vocabularies. In 2016, Seerainer and Sabutsch affirm: “Semantic interoperability within a nationwide electronic patient record, entailing the interconnection of highly diverse organizations with various IT systems, can only be achieved by providing standardized terminologies” (113) . Their paper (113) describes the development of a national terminology system to share EHR data in Austria. To achieve these, multiple terminologies are loaded onto a terminology server, partially translated, and made available for users along with a manual on how to implement and use the terminologies (113). It is obvious that an approach like this is very different from what Brochhausen et al. call computable semantics. While the standardized terminology restricts the number of terms used, the interpretation of what those terms mean is not achieved, in fact not even guided by the computer. The interpretation of the allowable terms and values is completely externalized to human agents using them. The manuals and textual definition might provide some insight into what the intended meanings are, but ultimately, there is no guarantee that the terms and values are interpreted in the same way from one user to the next and from one clinical site to the next. The inherent problem of interpreting terms from terminologies and vocabularies has been highlighted by three studies in the past, which show that inter- and intra-coder equivalence in coding medical material with SNOMED CT did not surpass 58% (90, 114, 115). It is important to note that those findings predate substantial changes in SNOMED CT, which moved it to be more like an ontology (116), while currently considerable parts of it lack both formal and textual definitions.

We have mentioned above the role that the interplay between informal terminology systems and ontologies plays for semantic integration in conjecture with ontologies (104). In creating that interplay, researchers and developers frequently rely on mapping one or more terminological resources with one or more ontologies.

## 6. Future opportunities

### 6.1. Linguistic opportunities

The use of NLP for processing clinical routine data has long been seen as a rather academic topic, which may be useful to solve well-delineated problems, but whose implementation within robust IT environments was still a long way away and not safe enough for clinical decision support. Whereas in the 20th century AI and NLP was characterized by unrealistic promises and several drawbacks, the universal AI boom of the last 10 years has brought intelligent applications including “understanding” of human language within user reach.

But the question of how these developments can be translated into real improvements of health care and health management is not ultimately answered, in particular, how can they be harnessed for events with high and urgent information and action needs, such as pandemics? A recent review (117) of approximately 150 NLP studies on COVID-19 focused on the extraction of information from published texts and identified the need for using clinical documents as a source as largely unmet.

Success criteria for the application of NLP in electronic patient records are crucial, for example user-friendly NLP platforms that implement current technology, which are extensible and customizable *via* open interfaces, and enable high quality text recognition, thus creating trust in their use and enabling automation of medical documentation processes. The performance of clinical NLP systems should be easily proven *via* benchmarks and shared tasks within the process of the adaptation to the application domain. This has to be done with a consistent and continuous application-oriented development of semantic standards, in particular ontologies such as SNOMED CT and information models such as HL7-FHIR, with specific consideration of the output of clinical NLP systems. This goes along with easy access to terminology resources, corpora and language models optimized for the clinical language. Modern hospital IT should therefore support computationally intensive AI processes, such as with GPU computers, and therefore enables easy integration and adaptation of NLP systems specifically but support multimodal approaches in general by combining, e.g., OMICS, image and textual resources widening the patient’s digital pheno- and genotype scope. Qualitatively adapted NLP systems, at its best, adapted to an entire target domain (118), can therefore be seen as one part in a holistic P5 medicine approach. The domain-adapted resources should not be locked in this setting, but legal and regulatory frameworks should facilitate the use and reuse of medical data to train artificial intelligence across institutions, as well as the sharing of domain-adapted NLP models.

### 6.2. Ontology-related opportunities

Over the last few years, we see a growing interest in using ontologies and SWTs. While there is still a communication and knowledge gap regarding computable semantics, ontologies get increasingly used. We have pointed out the two major repositories for biomedical ontologies, the BioPortal (85) and the OBO Foundry (79, 80). Having repositories making biomedical ontologies available which are typically focused on a specific domain or use case, raises the question of how expanding the coverage of those repositories, the individual classes and relations can be orchestrated and organized. As mentioned above, the strategy of the BioPortal and the OBO Foundry are quite different: the BioPortal is an open repository that allows developers to upload their ontology, then provide tools for users to identify the right ontology for their project. The OBO Foundry’s approach from its very beginning has been more coordinated. This is not only true regarding the principles submitted ontologies need to follow, but also the evolution towards more rigorous inclusion criteria (79, 80). Figure 3 shows the initial conception of OBO Foundry coverage regarding biological and biomedical domains and the axes the coverage was supposed to expand along. However, as the OBO Foundry grew, there have been an increasing number of ontologies added that cut across some of the axes shown in Figure 3. This meant that the orthogonality of the ontologies in the OBO Foundry was a work in progress in the initial years (119), but lately, independent analysis demonstrated the positive impact of OBO Foundry principles on the quality measure of OBO Foundry ontologies (120). Yet there are important biomedical ontologies growing independent of the OBO Foundry and uncoordinated with shared foundational ontologies. In the first place, this is the case with SNOMED CT as a resource that requires licensing, which contradicts the OBO Foundry principles. For the future, it is an important issue to ensure that the resources required for clinical interoperability are freely available to all participants as so-called knowledge commons. Since the development and maintenance of high quality semantic resources require considerable efforts, a strategy for sustainable evolution still has to be developed.

However, increasing unification of the existing ontologies in the OBO Foundry and principled expansion of its coverage might require further development and assessment of methodologies to guide ontology design decisions and representational strategies. The publication of ISO 23903 (5) *Interoperability and integration reference architecture—Model and framework*, marks one new opportunity to provide rigorous guidance for orchestrated ontology development. Brochhausen et al. have recently demonstrated the use of the reference architecture model and framework to analyze ontological representation and modeling for clinical data and other data relevant to biobanking for orthopedic trauma care (121). Viewing the data and specimen management as the business case represented in the *Business View* as defined by the GCM, necessary changes from the perspective of local EHR systems become obvious and can be handled on a principled basis. The local EHRs are likely to enforce one patient identifier (ID) per patient. The business case of integrating data from multiple healthcare providers requires transition to allowing multiple patient IDs per patient to accommodate sampling regarding tumor progression happening in multiple providers (121, 122) . This is modeled by progressing through the *Enterprise View*, allowing multiple IDs into *the Business View* (Figure 2), which provides ways to query for patients across multiple patient IDs. The result is the ontology design decision which allows a one-to-many relationship between patients and IDs. This certainly could have been done based on *ad hoc* decision, but the principled approach, if used consistently, will allow increased rigor on ontology design decisions.

They showed that the reference architecture assisted in the resolution of representational design decisions and provided a rigorous way of managing such representation questions. Additional research is needed to test the usefulness of ISO 23903 for this purpose and alternative methods for rigorous methods of ontology design need to be developed and evaluated.

## Data availability statement

The original contributions presented in the study are included in the article/supplementary material, further inquiries can be directed to the corresponding author.

## Author contributions

MK and SS wrote the sections on NLP. MB and CZ contributed to the section on ontologies, supported by SS. BB wrote the background section and contributed to the overall design of the article, as well as to Section 6 **Future opportunities**. All authors contributed to the article and approved the submitted version.

## Conflict of interest

The authors declare that the research was conducted in the absence of any commercial or financial relationships that could be construed as a potential conflict of interest.

## Publisher’s note

All claims expressed in this article are solely those of the authors and do not necessarily represent those of their affiliated organizations, or those of the publisher, the editors and the reviewers. Any product that may be evaluated in this article, or claim that may be made by its manufacturer, is not guaranteed or endorsed by the publisher.
